# Editorial: Unswitched memory B cells in human health and disease

**DOI:** 10.3389/fimmu.2024.1455243

**Published:** 2024-07-22

**Authors:** Moriah. J. Castleman

**Affiliations:** Basic Immunology Branch, Division of Allergy, Immunology & Transplantation (DAIT), National Institute of Allergy & Infectious Disease (NIAID), Rockville, MD, United States

**Keywords:** unswitched memory B cells, viral infection, autoimmune disease, marginal zone (MZ) B cell, IgM+ B cells, IgD+ B cells, germinal center

The focus of this special Research Topic is unswitched memory B cells in human health and disease. Unswitched memory B cells are antigen experienced cells expressing the traditional memory marker CD27 and the default IgM/IgD immunoglobulins thus have not class switched to other immunoglobulin isotypes. This terminology primarily refers to a B cell subset expressing both IgM and IgD (CD27+IgM+IgD+), followed by a smaller subset of ‘IgD only’ cells (CD27+IgM-IgD+) and occasionally includes the ‘pre-switched’ memory subset expressing only IgM (CD27+IgM+IgD-). The heterogeneity of the unswitched memory B cell population is reflected in the numerous alternative names found in the literature (e.g. non-switched memory, circulating marginal zone, marginal zone-like), contributing to the complexity of defining their function in humoral immunity. This editorial summarizes the articles that contributed to this special Research Topic.

The origin of unswitched memory B cells in humoral immunity is controversial since these cells display features of antigen experience through somatic hypermutation yet retain the default immunoglobulin isotype classes IgM/IgD. However, Kuppers and colleagues make a case that unswitched memory B cells in adults are derived from the germinal center (GC) reaction as opposed to T-independent responses or antigen-independent prediversified pathways. The investigators lay out a convincing argument focusing on the similarity between unswitched and switched memory B cells including evidence of shared clonal lineages, gene expression patterns and antigen-specific cells that arise from viral T-dependent humoral responses. Importantly, they note that unswitched memory B cells carry a substantial level of somatic hypermutation and many of these cells have mutated BCL6 genes, which only arises via high levels of mutation in GC reactions. Altogether, this commentary is thought-provoking and provides an excellent summary of the history of unswitched memory B cells in humans.

Yuuki et al. review BCR repertoire analysis in the context of autoimmune diseases studies and the insights gained about unswitched memory B cells. Specifically, they comment on biased IGHV gene usage and longer CDR3 length in unswitched memory B cells in SLE patients compared to healthy controls and argue that a subset of unswitched memory B cells may arise through the extrafollicular pathway which is known to be activated in autoimmune disease. Together, this review provides an alternative perspective on unswitched memory B cells in autoimmune disease.

Gómez et al. examined unswitched memory B cells in PBMCs from female patients with Systemic Lupus Erythematosus (SLE) compared to healthy controls in Mexico. These investigators showed a dysregulation of the BAFF system in SLE patients. Specifically, there was a higher frequency of unswitched memory B cells expressing BCMA (B cell maturation antigen), one of the BAFF receptors, along with a higher frequency of cells with membrane bound BAFF compared to healthy controls. Thorough examination of other populations including double negative, naive and switched memory B cells identified other signs of BAFF system dysregulation in SLE patients. Interestingly, examination of the surface markers typically associated with atypical B cells (CXCR5, CD11c) revealed a reduced frequency of the CXCR5+CD11c- unswitched memory B cell subset with an increased frequency of the CXCR5-CD11c+ subset. Intriguingly, the investigators find a negative correlation between the frequency of the unswitched memory B cell subset expressing CD11c with expression levels of BAFFR. Overall, this study highlights the link between the BAFF system and its impact on unswitched memory B cells in human autoimmune disease.

Torres and colleagues examined unswitched memory B cells in the context of infectious disease. Investigation into unswitched memory B cells in COVID-19 revealed a negative correlation between increasing levels of systemic TNFα and decreasing frequency of this cell population in severe SARS-CoV-2 infection. Intriguingly, one subset of unswitched memory B cells that only express IgD (CD27+IgM-IgD+) was increased with severe viral infection and levels correlated with increased titers of autoreactive antibodies. Phenotypically, unswitched memory B cells displayed higher CD86 expression and loss of CD21 (indicative of activation) with lower levels of the inhibitory receptor CD72 in severe viral infection. Further, unswitched memory B cells also displayed enhanced BCR signaling capacity compared to switched memory B cells and were able to express IL-6 and TNF-α in response to stimulation with TLR7/8 or TLR9 viral ligands. In total, this study provides insights into the multifaceted role of unswitched memory B cells during viral infection.

Since many different types of B cells exist in homeostasis, arise in response to infection, or develop along with autoimmunity and strategies used in the field to identify subsets can be heterogeneous, Pernes et al. caution against using surface markers alone to profile B cell subsets. These investigators recommend using a combination of surface markers and transcriptome as captured by CITE-sequencing technologies; whereby traditional flow-cytometric gating strategies can be applied to sequencing data for reads of oligo-tagged antibodies to surface markers on B cell populations. With advances in profiling protein expression via sequencing and increased accessibility of single-cell sequencing platforms, CITE-seq is now possible for many research groups and may more accurately identify differing B cell subsets. However, this editor believes that development of technology that can link protein & gene expression with cellular function will ultimately provide the best advantage to shedding light on the complex nature of unswitched memory B cells in human health and disease.

Although this special Research Topic identified novel roles of unswitched memory B cells in autoimmunity and viral infection and stimulated thoughtful commentary on the origin of unswitched memory B cells, much remains to be learned about this enigmatic population in human health and disease.

## Author contributions

MC: Writing – review & editing, Writing – original draft, Conceptualization.

